# A note on a generalized single step theory for any number of hierarchical genomic matrices

**DOI:** 10.1186/s12711-026-01084-3

**Published:** 2026-09-14

**Authors:** Miguel Pérez-Enciso

**Affiliations:** 1 Centre for Research in Agricultural Genomics (CRAG), CSIC-IRTA-UAB-UB Consortium, Bellaterra, Barcelona, 08193 Spain; 2 ICREA, Passeig de Lluís Companys 23, Barcelona, 08010 Spain

## Abstract

**Background:**

The Single Step algorithm allows combining information from genotyped and un-genotyped individuals, provided they are connected by a pedigree. However, current single step theory is limited to a single list of markers.

**Results:**

We present a generalized single step (GSS) method that can accommodate any number of hierarchical molecular datasets (e.g. sequence, high and low density arrays) and pedigree, avoiding imputation. We prove that a similar efficient inversion algorithm exists. The method is recursive, starting with the highest marker density scenario. We illustrate the method with simulation and show that GSS can increase predictive accuracy compared to standard single step. R code is provided so that custom scenarios can be easily compared, either with simulated or real data.

**Conclusion:**

The method developed generalizes extant single step theory to any number of hierarchical molecular relationship matrices, broadening the scenarios where single step can be applied. A topic of particular interest can be ecology field data or human populations where pedigree is not available, but where samples sequenced and genotyped at different densities can exist. GSS can also be a useful tool to optimize allocation of genotyping and / or sequencing resources.

## Background

The single step (SS) approach [1, 2] was proposed to include data from genotyped and ungenotyped individuals in genomic selection while avoiding collapsing information from ungenotyped individuals into some sort of ‘pseudo-phenotypes’ such as daughter yield deviations or other adjustments (see references e.g. in Legarra et al. [3]). The SS methodology allows us to combine seamlessly information from both genotyped and ungenotyped individuals, provided these individuals are connected by pedigree. A most convenient advantage of SS is that it requires the inverse of a matrix the size only the number of individuals genotyped, which is usually much smaller than the total number of individuals in the pedigree. Due to its advantages over competing approaches, SS is the method of choice in numerous practical genomic selection (GS) breeding schemes [3, 4] and efficient software is available [5].

Current SS theory is limited to a single list of markers and therefore individuals are partitioned in two groups only, those genotyped and ungenotyped. Very often, though, SNP arrays of different densities and even up to complete sequence data can coexist in a single breeding program. Available SS theory cannot be applied to this scenario, and the current practice in this scenario is to impute up to the highest marker density list.

Akdemir et al. [6] proposed a method for combining partially overlapping genomic relationship matrices. Their method builds on completely different principles than SS. It is an iterative method that carries out imputation using an EM algorithm and capitalizes on the fact that co-variance matrices follow a Wishart distribution. In a related vein, Christensen et al. [7] presented an approach to combine heterogeneous sources of information, using a two-step SS approach.

In all, it would be desirable to have a general SS theory that can deal with heterogeneous SNP data sources, since individuals are often genotyped with a variety of arrays of different densities and SS is the method of choice for genomic prediction in the industry. The purpose of this short note is to develop a recursive generalized single step (GSS) approach that can incorporate any number of hierarchical marker datasets, such as sequence and arrays of increasing density. We prove that the same inversion algorithm as for standard SS holds. It avoids the two-step procedure of imputation followed by genetic evaluation and is faster than other iterative methods [6]. We illustrate the theory with a simple simulation example and provide documented R code in https://github.com/miguelperezenciso/GSS.

## Materials and methods

### Theory

In the following, we assume the usual linear mixed model:

*y = Xb + Wu + e,*

where **y** contains the phenotypes, **b** is the fixed effects vector, random genetic values are in **u**, residuals in **e**, and **X** and **W** are incidence matrices. Single Step theory considers a subset **u**_1_ in **u** comprising individuals without molecular data and subset **u**_2_ which have been genotyped. Both **u**_1_ and **u**_2_ are related through a common pedigree (**P**) that results in numerator relationship matrix **A**. When marker information on *m* SNPs is available, a genomic relationship matrix (GRM) **G** can be computed as

1 $${\bf{G}} = {{\left( {{\bf{M}} - 2p} \right){{\left( {{\bf{M}} - 2{\bf{p}}} \right)}^\prime }} \over {2{\bf{p{^{\prime}}}}\left( {1 - {\bf{p}}} \right)}},$$

[8] where **M** is an *n* individual by *m* marker matrix containing genotypes (coded as 0,1,2) and **p**, a *m*-dimension vector with corresponding allele frequencies. Legarra et al. (2009) and Christensen and Lund (2010) showed that the variance of breeding values can be approximated by

2 $$\begin{aligned} \mathbf{H} &= Var \left( \begin{array} {l}\boldsymbol{u}_1 \\ \boldsymbol{u}_2 \end{array} \middle| \mathbf{P}, \mathbf{M}, \dots \right) \\ &= \begin{pmatrix} \mathbf{H}_{11} & \mathbf{H}_{12} \\ \mathbf{H}_{21} & \mathbf{H}_{22} \\ \end{pmatrix} \sigma_u^2 \\ &\sim \begin{pmatrix} \mathbf{A}_{11} - \mathbf{A}_{12}\mathbf{A}_{22}^{-1}\mathbf{A}_{21} + \mathbf{A}_{12}\mathbf{A}_{22}^{-1}\mathbf{G}_{22 }\mathbf{A}_{22}^{-1}\mathbf{A}_{21} & \mathbf{A}_{12}\mathbf{A}_{22}^{-1}\mathbf{G}_{22} \\ \mathbf{A}_{21}\mathbf{A}_{22}^{-1}\mathbf{G}_{22} & \mathbf{G}_{22} \end{pmatrix} \sigma_u^2 \end{aligned}$$

Above, subindices *ij* refer to the block *ij* of the given matrix, *i* = 1 refers to ungenotyped individuals and *i* = 2, to genotyped ones. For instance, **A**_12_ is the numerator relationship block submatrix between genotyped and ungenotyped individuals. In the case of **G**_**22**_, subscript is technically redundant since only individuals in block 2 are genotyped, **G** = **G**_**22**_, e.g., **G**_**11**_ is not defined. However, we prefer to keep the subscript to make notation consistent when generalizing to any number of marker lists.

Equation (2) is obtained after some simplifications. In particular, it is assumed that (i) marker effects can be modeled as quantitative variables that follow a multivariate normal distribution; (ii) information contained in markers ‘dominates’ over pedigree for genotyped individuals, i.e., p(**u**_2_**|M**, **P**) ~ p(**u**_2_**|M**); and (iii) marker allele frequencies computed from current data (**p**) are unbiased estimates of those of the base population [3, 9].

In this work, we generalize SS current theory to any number of hierarchical SNP datasets. Initially, without loss of generality, let us consider three subsets: no molecular information (only **P** available), SNP array (**M**) and sequence (**M**_**S**_) information. We consider that sequence is available only for a subset **u**_3_ of those individuals that are also genotyped with the array, **u**_***2***_. We further assume that all markers in **M** are also present in **M**_**S**_, and that individuals are connected through a pedigree **P**. Therefore, the goal is to obtain:

$$Var\left( {\begin{array}{*{20}{c}}{{{\mathbf{u}}_1}} \\ {{{\mathbf{u}}_2}} \\ {{{\mathbf{u}}_3}} \end{array}\left| {{\mathbf{P}},{\mathbf{M}},{{\mathbf{M}}_S}, \ldots } \right.} \right) = \left( {\begin{array}{*{20}{c}}{{H_{11}}}&{{H_{12}}}&{{H_{13}}} \\ {{H_{21}}}&{{H_{22}}}&{{H_{23}}} \\ {{H_{31}}}&{{H_{32}}}&{{H_{33}}} \end{array}} \right)\sigma _u^2 = {\mathbf{H}}\sigma _u^2$$

assuming that the expectation for all breeding values in **u** is zero. As with SS theory, once the generalized **H** is derived, standard mixed model theory can be employed for prediction. First, we note that

$${H_{33}} = Var({{\bf{u}}_3}|{\bf{P}},{\bf{M}},{{\bf{M}}_s}) \sim Var({{\bf{u}}_3}|{{\bf{M}}_s}) = {\bf{S}}\sigma _u^2,$$

where **S** is the genomic relationship matrix computed applying Eq. (1) to sequence data (or the highest density SNP array available). The equation above means that available sequence information makes the contribution from the array and that of the pedigree ignorable, i.e., the same assumption as in standard SS. Next, following the same reasoning as in single step theory (Eq. 2), except that **G** is used instead of **A**, and **S** instead of **G**:

$$\begin{aligned}\mathbf{H}_{22} &= Var(\boldsymbol{u}_2 | \mathbf{P}, \mathbf{M}, \mathbf{M}_S) \\ &\sim Var(\boldsymbol{u}_2 | \mathbf{M}, \mathbf{M}_S) \\ &= (\mathbf{G}_{22} - \mathbf{G}_{23} \mathbf{G}_{33}^{-1} \mathbf{G}_{32} + \mathbf{G}_{23} \mathbf{G}_{33}^{-1} \mathbf{S} \mathbf{G}_{33}^{-1} \mathbf{G}_{32}) \sigma_u^2,\end{aligned}$$

and by the same token:

$$\begin{aligned} {H_{23}} &= Cov\left( {{{\bf{u}}_2},{{\bf{u}}_3}|{\bf{P}},{\bf{M}},{{\bf{M}}_s}} \right) \\ &\sim Cov\left( {{{\bf{u}}_2},{{\bf{u}}_3}|{\bf{M}},{{\bf{M}}_s}} \right) = {{\bf{G}}_{23}}{\mkern 1mu} {\bf{G}}_{33}^{ - 1}{\bf{S}}\sigma _u^2\end{aligned}$$

Next, it should be noted that (see Section 3.2 in [3]):

3 $$\begin{aligned} f(\mathbf{u}_1 | \mathbf{u}_2, \mathbf{u}_3) &=\ N\left[ \begin{pmatrix} \mathbf{A}_{12} & \mathbf{A}_{13} \end{pmatrix} \begin{pmatrix} \mathbf{A}_{22} & \mathbf{A}_{23} \\ \mathbf{A}_{32} & \mathbf{A}_{33} \end{pmatrix}^{-1} \begin{pmatrix} \mathbf{u}_2 \\ \mathbf{u}_3 \end{pmatrix}, \right. \\ & \quad \left. \left\{ \mathbf{A}_{11} - \begin{pmatrix} \mathbf{A}_{12} & \mathbf{A}_{13} \end{pmatrix} \begin{pmatrix} \mathbf{A}_{22} & \mathbf{A}_{23} \\ \mathbf{A}_{32} & \mathbf{A}_{33} \end{pmatrix}^{-1} \begin{pmatrix} \mathbf{A}_{21} \\ \mathbf{A}_{31} \end{pmatrix} \right\} \sigma_u^2 \right] \end{aligned}$$

Now let us take the submatrix of **H** that contains the joint variance of **u**_2_ and **u**_3_, i.e., those individuals with molecular data, either sequence or SNP array:

4 $$Var\left( {\matrix{ {{{\bf{u}}_2}} \cr {{{\bf{u}}_3}} \cr } {\rm{|}}{\bf{P}},{\bf{M}},{{\bf{M}}_S}, \ldots } \right) = \left( {\matrix{ {{H_{22}}} & {{H_{23}}} \cr {{H_{32}}} & {{H_{33}}} \cr } } \right)\sigma _u^2$$

From above, integrating out (**u**_2_, **u**_3_) in (3) using (4), it follows that

$$\begin{aligned} \mathbf{H}_{11} &= Var(u_1 | \mathbf{P}, \mathbf{M}, \mathbf{M}_S) \\\\ &= \left[ \mathbf{A}_{11} - \begin{pmatrix} \mathbf{A}_{12} & \mathbf{A}_{13} \end{pmatrix} \begin{pmatrix} \mathbf{A}_{22} & \mathbf{A}_{23} \\\\ \mathbf{A}_{32} & \mathbf{A}_{33} \end{pmatrix}^{-1} \begin{pmatrix} \mathbf{A}_{21} \\\\ \mathbf{A}_{31} \end{pmatrix} \right] \sigma_u^2 \\\\ &+ \left[ \begin{pmatrix} \mathbf{A}_{12} & \mathbf{A}_{13} \end{pmatrix} \begin{pmatrix} \mathbf{A}_{22} & \mathbf{A}_{23} \\\\ \mathbf{A}_{32} & \mathbf{A}_{33} \end{pmatrix}^{-1} \begin{pmatrix} \mathbf{H}_{22} & \mathbf{H}_{23} \\\\ \mathbf{H}_{32} & \mathbf{H}_{33} \end{pmatrix} \begin{pmatrix} \mathbf{A}_{22} & \mathbf{A}_{23} \\\\ \mathbf{A}_{32} & \mathbf{A}_{33} \end{pmatrix}^{-1} \begin{pmatrix} \mathbf{A}_{21} \\\\ \mathbf{A}_{31} \end{pmatrix} \right] \sigma_u^2 \end{aligned}$$

and

$$\begin{aligned}\mathbf{H}_{1,i>1}&= \begin{pmatrix} \mathbf{H}_{12} & \mathbf{H}_{13} \end{pmatrix}= \operatorname{Cov}\left(\mathbf{u}_1,[\mathbf{u}_2,\mathbf{u}_3]' \mid \mathbf{P},\mathbf{M},\mathbf{M}_S\right) \\[6pt]&= \begin{pmatrix} \mathbf{A}_{12} & \mathbf{A}_{13} \end{pmatrix}\begin{pmatrix}\mathbf{A}_{22} & \mathbf{A}_{23} \\\mathbf{A}_{32} & \mathbf{A}_{33}\end{pmatrix}^{-1}\begin{pmatrix}\mathbf{H}_{22} & \mathbf{H}_{23} \\\mathbf{H}_{32} & \mathbf{H}_{33}\end{pmatrix}\sigma_u^2\end{aligned}$$

Because the generalized **H** matrix has the same structure as in the standard SS theory, its inverse is also given by

5 $${H^{ - 1}} = {{\mathbf{A}}^{ - 1}} + \left( {\begin{array}{*{20}{c}}0&0 \\ 0&{{{\left[ {\begin{array}{*{20}{c}}{{{\mathbf{H}}_{22}}}&{{{\mathbf{H}}_{23}}} \\ {{{\mathbf{H}}_{32}}}&{{{\mathbf{H}}_{33}}} \end{array}} \right]}^{ - 1}} - {{\left[ {\begin{array}{*{20}{c}}{{{\mathbf{A}}_{22}}}&{{{\mathbf{A}}_{23}}} \\ {{{\mathbf{A}}_{32}}}&{{{\mathbf{A}}_{33}}} \end{array}} \right]}^{ - 1}}} \end{array}} \right)$$

which requires the inverse of matrices with maximum dimension the number of individuals with molecular data. Equation (5) is shown to hold in R and Rmd scripts available at https://github.com/miguelperezenciso/GSS.

**Fig. 1:**
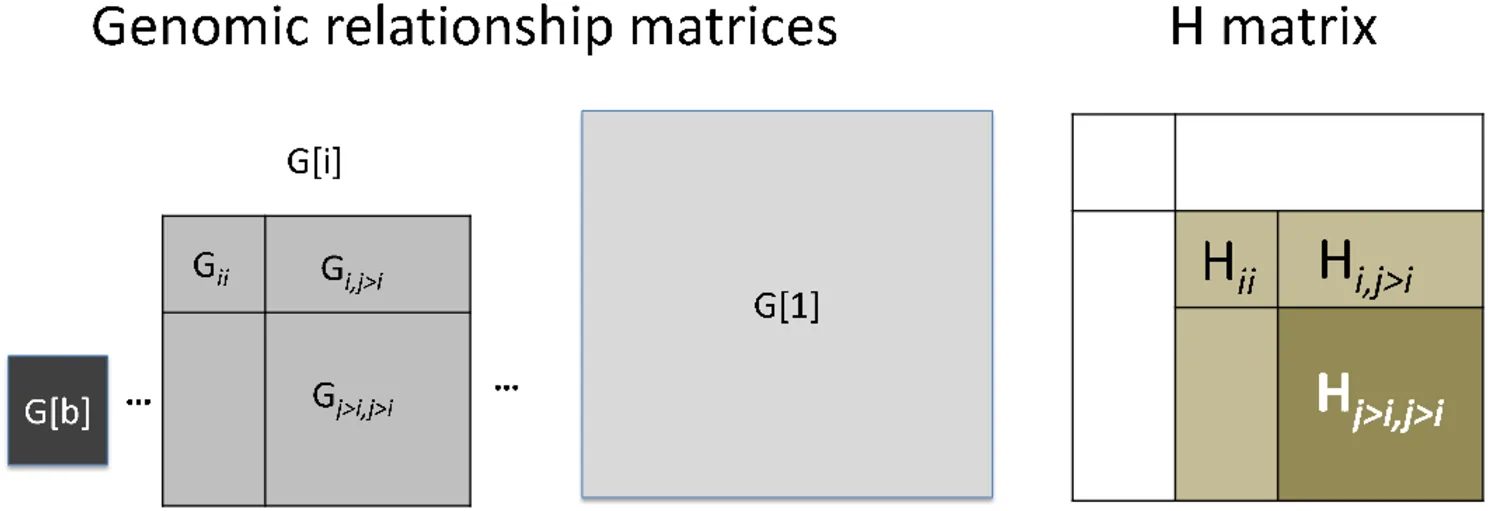
Graphical representation of matrices involved in obtaining H in the generalized single step approach. Legend: It is assumed that molecular information is arranged in a series of *b* ordered hierarchical marker sets (e.g., sequence, high density array, low density array) down to pedigree. Considering that set *b* is the one with highest marker density, it is also assumed that all individuals in set *i* are also in sets *j < i* and that all markers in set *i* are in sets *j > i*. A molecular relationship matrix **G**_[i]_ can be computed for each marker set *i*, such that dim(G_[i]_) >dim(G_[j]_) for all *j < i*. **H** is computed recursively backwards starting with the last and highest marker density block **H**_bb_ = **G**_[b]_. The first matrix G _[1]_ is usually the numerator relationship matrix, which can be obtained efficiently from the pedigree using the tabular method. In i-th step, to obtain the **H**_ii_ and **H**_i,j > i_ submatrices, Eqs. (5) and (6) are applied, which in turn require submatrices **G**_[i]ii_, **G**_[i]i,j > i_, **G**_[i]j > i,j > i_ and **H**_j > i,j > i_. Submatrix **G**_[i]ii_ is that corresponding to individuals genotyped with i-th marker set but not with higher density SNP sets; **G**_[i]j > i,j > i_ submatrix corresponds to those individuals genotyped also for higher density sets than that in i-th set, and **H**_j > i,j > i_ is the current **H** matrix, before information from **G**_[i]_ is utilized

Importantly, theory above lends itself to generalize to any number of marker datasets, as long as they are hierarchical. Suppose we a have list of *b* marker datasets (blocks) with covariance matrices **G**_[1]_, **G**_[2]_, … **G**_[b]_, such that block 1 corresponds to ungenotyped individuals and block *b*, to those with highest genotyped density; in the case above **G**_[1]_ = **A**, and **G**_[3]_ = **S**. Then for any i-th block (Fig. 1):

6 $$\begin{aligned} \mathbf{H}_{ii} &= \Bigl[ \mathbf{G}_{[i]ii} - \mathbf{G}_{[i]i,j>i} \mathbf{G}_{[i]j>i,j>i}^{-1} \mathbf{G}_{[i]j>i,i} \\ &\qquad{} + \mathbf{G}_{[i]i,j>i} \mathbf{G}_{[i]j>i,j>i}^{-1} \mathbf{H}_{j>i,j>i} \mathbf{G}_{[i]j>i,j>i}^{-1} \mathbf{G}_{[i]j>i,i} \Bigr]\sigma_u^2 . \end{aligned}$$

and

7 $${H_{i,j > i}} = \left[ {{{\bf{G}}_{\left[ i \right]i,j > i}}{\bf{G}}_{\left[ i \right]j > i,j > i}^{ - 1}{H_{j > i,j > i}}} \right]\sigma _u^2,$$

The key is to start backwards from the highest density SNP list as in the original SS algorithm and where block diagonals **H**_**ii**_ and off-diagonal blocks **H**_**i,j > i**_ are computed using the same principles as in standard SS. The algorithm can be specified as follows:

1. Initialize **H** matrix with dimension total number of individuals
2. Set ${{\bf{H}}_{bb}} = {{\bf{G}}_{bb}}\sigma _u^2$, the last diagonal block in **H**
3. Proceed backwards, *i = b-1, … 1*
  a Obtain the genetic covariances of individuals in that block that are not in upper blocks ${{\bf{H}}_{ii}}$ from Eq. 6.
  b Obtain covariances between individuals in upper blocks and those only in current block ${{\bf{H}}_{i,\left\{ {i + 1, \ldots ,b} \right\}}}$ from Eq. 7
  c Equations 6 and 7 depend only on previously computed **H** blocks and on the GRM of current block, **G**_[**i**]_.

See Figure 1 and doHgss function in https://github.com/miguelperezenciso/GSS/blob/main/GSS.R.

As noted, Eqs. (6) and (7) are computed recursively, starting with ${\bf{H}_{bb}}{\rm{}} = {\bf{G}}_{bb}\sigma _u^2$, the individuals with highest marker density, and can be applied to any number of hierarchical marker sets. However, note that each step requires the inverse of submatrix ${\bf{G}}_{j > i,j > i}^{}$ (Eq. 6), with order of magnitude the sum of individuals in each of the blocks *i+1* to *b*. Therefore, having many blocks of large sizes can be computationally demanding. Further the inverse of **H** can also be obtained from

7 $${{\bf{H}}^{ - 1}} = {\bf{G}}_{\left[ 1 \right]}^{ - 1} + {\left( {\matrix{ 0 & 0 \cr 0 & {{\bf{H}}_{i > 1,i > 1}^{ - 1} - {\bf{G}}_{\left[ 1 \right]i > 1,j > 1}^{ - 1}} \cr } } \right)^{ - 1}},$$

where subscript *‘j > i*’ refers to matrix block with indices *i+1* to *b*. As in classical SS, GSS is optimal computationally when the first block, made-up of ungenotyped individuals, (**G**_1_) is the largest and equal to **A**, since its inverse can be computed in linear time and is sparse. Code in https://github.com/miguelperezenciso/GSS shows that Eq. (7) provides the actual inverse of generalized **H**. Note from Eq. (7) that a recursive argument can also be used to compute the inverse of **H**, since ${\bf{H}}_{i > 1,i > 1}^{ - 1}$ requires only inverting a matrix of the lower block dimension. However, gain in efficiency can be small since molecular relationship matrices cannot be inverted with efficient algorithms as for the numerator relationship matrix. Nevertheless, Misztal’s APY algorithm or equivalent algorithms can be used [10].

### Simulation

We illustrate the method with a simple simulation using AlphasimR [11]. AlphasimR uses a combined coalescence – gene dropping method as originally proposed in ms2gs software [12]. Specifically, both ms2gs and AlphasimR use the efficient Markovian Coalescent Simulator (MaCS [13]), to simulate the founder population.

Here, we simulated a founder diploid population of 1000 individuals with default AlphasimR coalescence parameters and a genome made up of 20 chromosomes with 1000 markers per chromosome. A quantitative trait with heritability h^2^ = 0.5 and controlled by 100 QTL (five per chromosome) was simulated. The simulation was run for three non - overlapping generations, 1000 random matings per generation produced one offspring per mating, making a total of 4000 individuals in the whole pedigree, including the founder individuals. The target was to predict breeding values of the 1000 individuals from the last (third) generation, where phenotypes were not available. To test the GSS algorithm, it was assumed that individuals of generation 1 and 2 were genotyped with 1000 random markers to mimic a medium density array and that generation 3 was fully ‘sequenced’, i.e., all 20,000 marker genotypes were known. The founder population (generation 0) was not genotyped.

Four strategies were compared: (i) no marker data available, only pedigree; (ii) pedigree plus low-density genotyping in generations 1–3 was available, i.e., standard SS; (iii) in addition, sequence data was available in generation 3, i.e., GSS; and (iv) all individuals, including founders, sequenced.

The theory proposed assumes marker lists are hierarchical. This is not necessarily so in all scenarios. As an illustration, we evaluated a scenario with two non-hierarchical arrays: a low-density array (1000 SNPs) and a high-density array (19,500 SNPs); 500 SNPs were shared between the two arrays. GRMs were obtained with the ‘exact’ EM-Wishart method in [6] or with GSS assuming that the two SNP arrays were truly hierarchical. Given that the EM-Wishart method was much slower than GSS (>100× slower) we run the same breeding structure as above but with 100 individuals per generation, instead of 1000.

The script available in GitHub (https://github.com/miguelperezenciso/GSS) easily allows modifying any of the parameters and scenarios studied here.

## Results and discussion

**Fig. 2:**
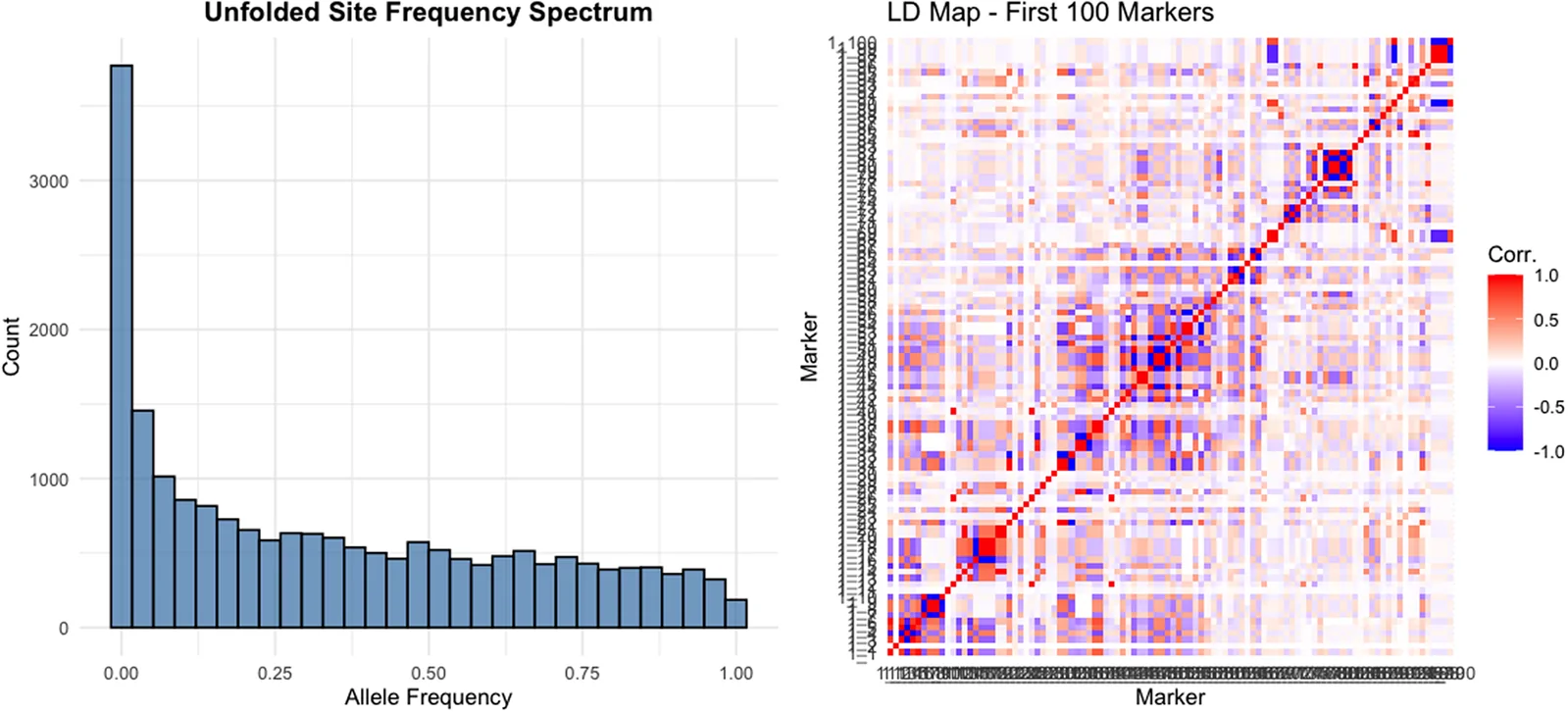
Unfolded site frequency spectrum of the ancestral allele in the whole pedigree (left panel) and correlation between genotypes of all pairs of first 100 markers (right panel). Legend: The sign of correlation is arbitrary

The purpose of this short note was to present the theoretical basis of a generalized single step method, rather than systematically evaluate its performance. As with any genomic prediction method, its advantage will depend on the specific scenario, and we provide documented R code that can be used for a customized analysis. Here, we evaluated the performance of GSS and related combined approaches in a relatively simple simulated pedigree. To verify plausibility of simulation, Fig. 2 shows that the simulated allele frequencies are enriched in low frequency variants and that linkage disequilibrium tends to be higher between close markers, as population genetics theory dictates [14].

**Fig. 3:**
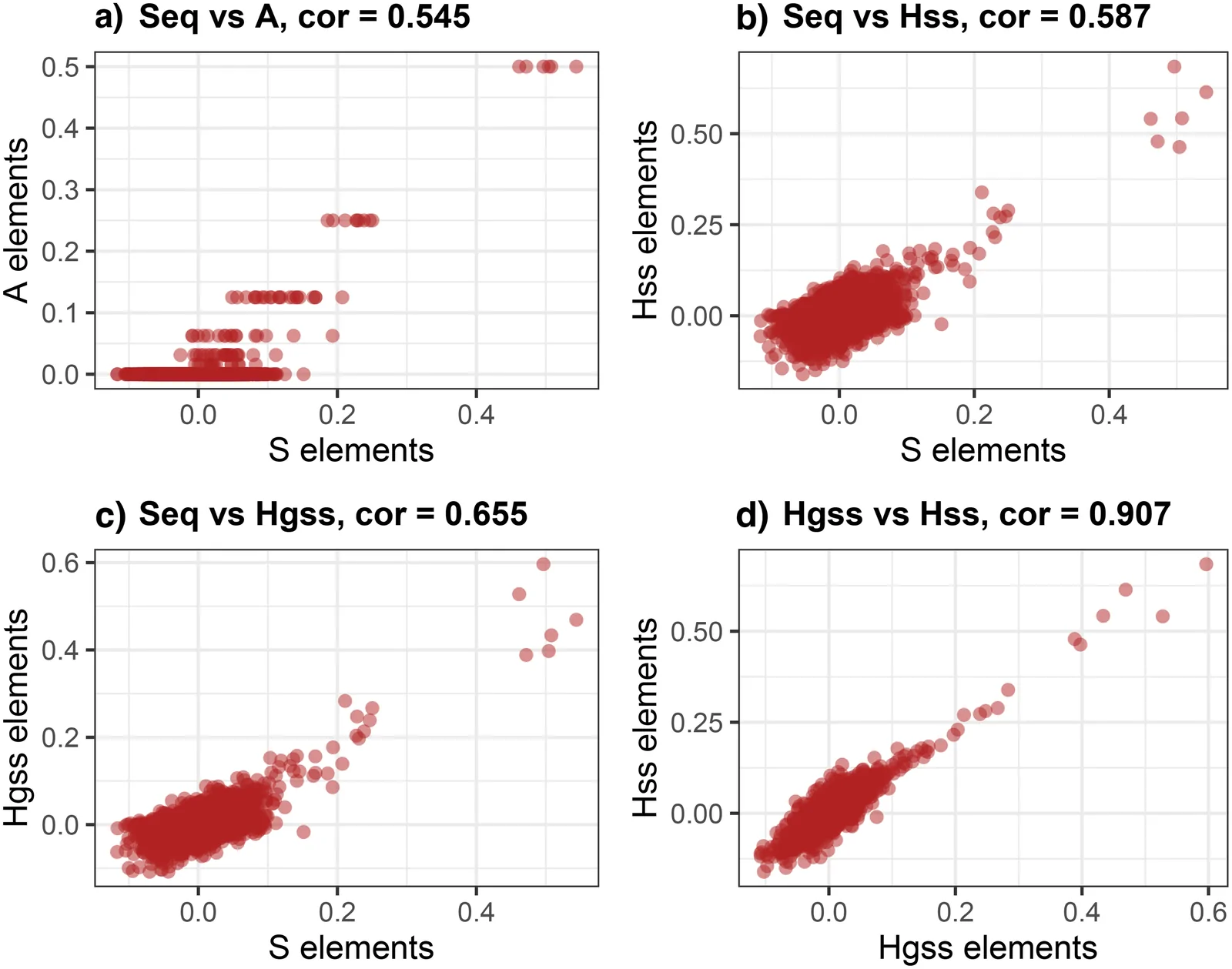
Correlations from 5000 random off-diagonal elements between different relationship matrices. Legend: A: numerator relationship matrix; S: sequence based GRM; Hss: standard single step matrix where founder individuals are ungenotyped and the remaining individuals are genotyped with 1k SNP array; and Hgss: generalized single step matrix where founder individuals are ungenotyped, generations 1–2 are genotyped with 1k array and generation 3 individuals are ‘sequenced’ (20k markers). Cor indicates correlation across the 5000 elements. Note the pattern in pedigree-based matrix, which depends on individuals’ generations (panel a)

Assuming that the ‘true’ relationship matrix would be that obtained when all markers from all individuals are used (**S**), Fig. 3 shows the relationship between **S**, different GRMs and numerator relationship matrix (**A**). Logically, the lowest relationship was between **S** and **A** matrices (Fig. 3a), where markers allow distinguishing relationships between founder individuals, assigned a 0 relationship from pedigree. As Fig. 3 shows, **A** coefficients are discrete and show uniform levels of relationship that are continuous when markers are utilized. While elements of SS and GSS **H** matrices are highly correlated, we observed a higher correlation between **S** and GSS **H** (Fig. 3c) than between **S** and standard SS **H** (Fig. 3b), reflecting that the amount of molecular data is larger in GSS than in the SS scenario.

**Fig. 4:**
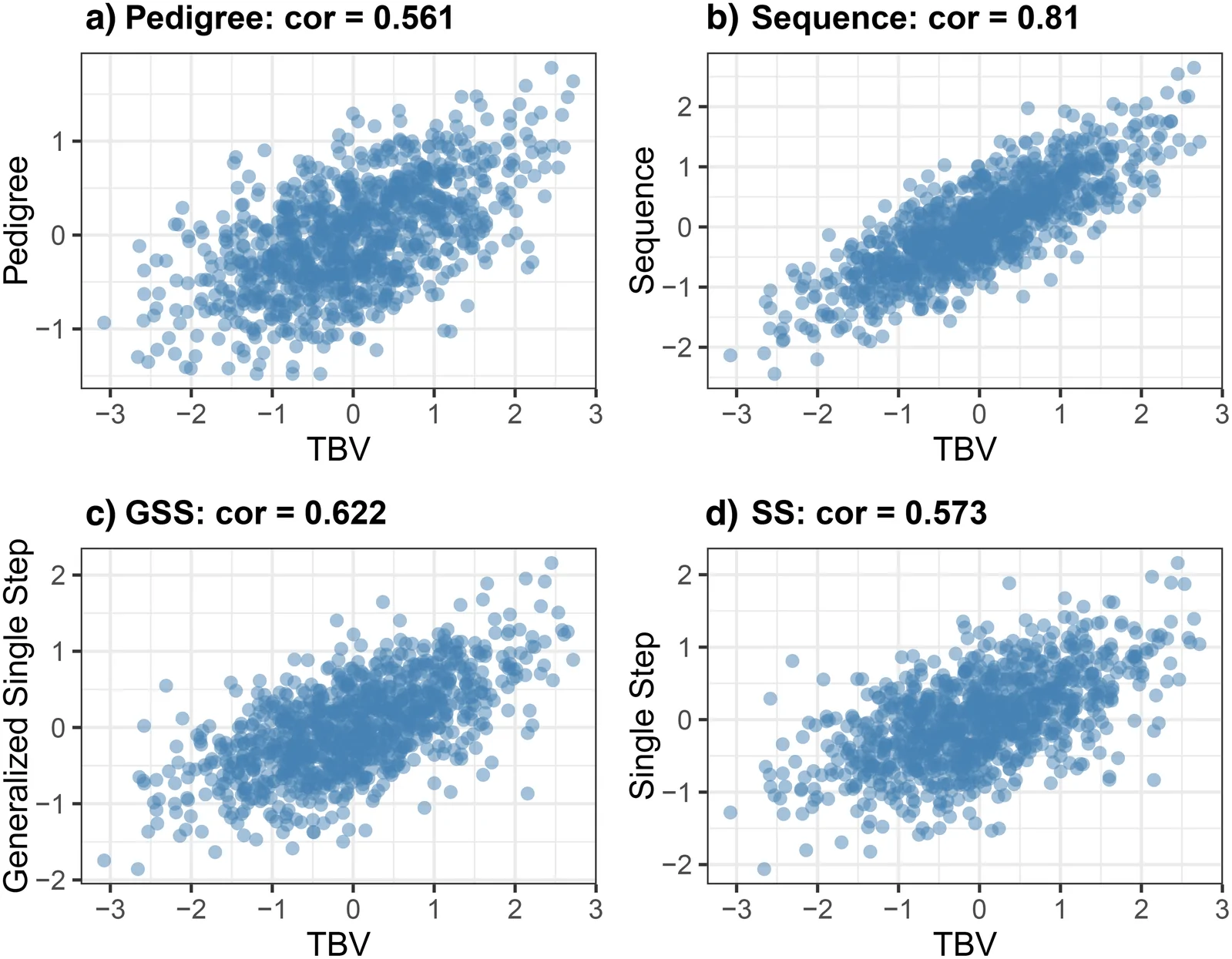
Relation between true breeding (TBV) values and several prediction methods for the 1000 non phenotyped individuals from last generation. Legend: (**a**) Only pedigree used; (**b**) All individuals sequenced; (**c**) Generalized single step (GSS): founders ungenotyped, generations 1 and 2 genotyped with 5k SNPs, and generation 3 sequenced; (**d**) Single step (SS): founders ungenotyped, generations 1–3 genotyped with 5k SNPs

In agreement with this, GSS allows an increase in predictive accuracy compared to standard SS (Fig. 4c vs. d). The increase in the scenario considered is moderate but actual improvements will depend on the specific parameters and will vary with replicate. Also as expected, prediction was worst without molecular data (Fig. 4a) and best with complete sequence in the whole population (Fig. 4b).

**Fig. 5:**
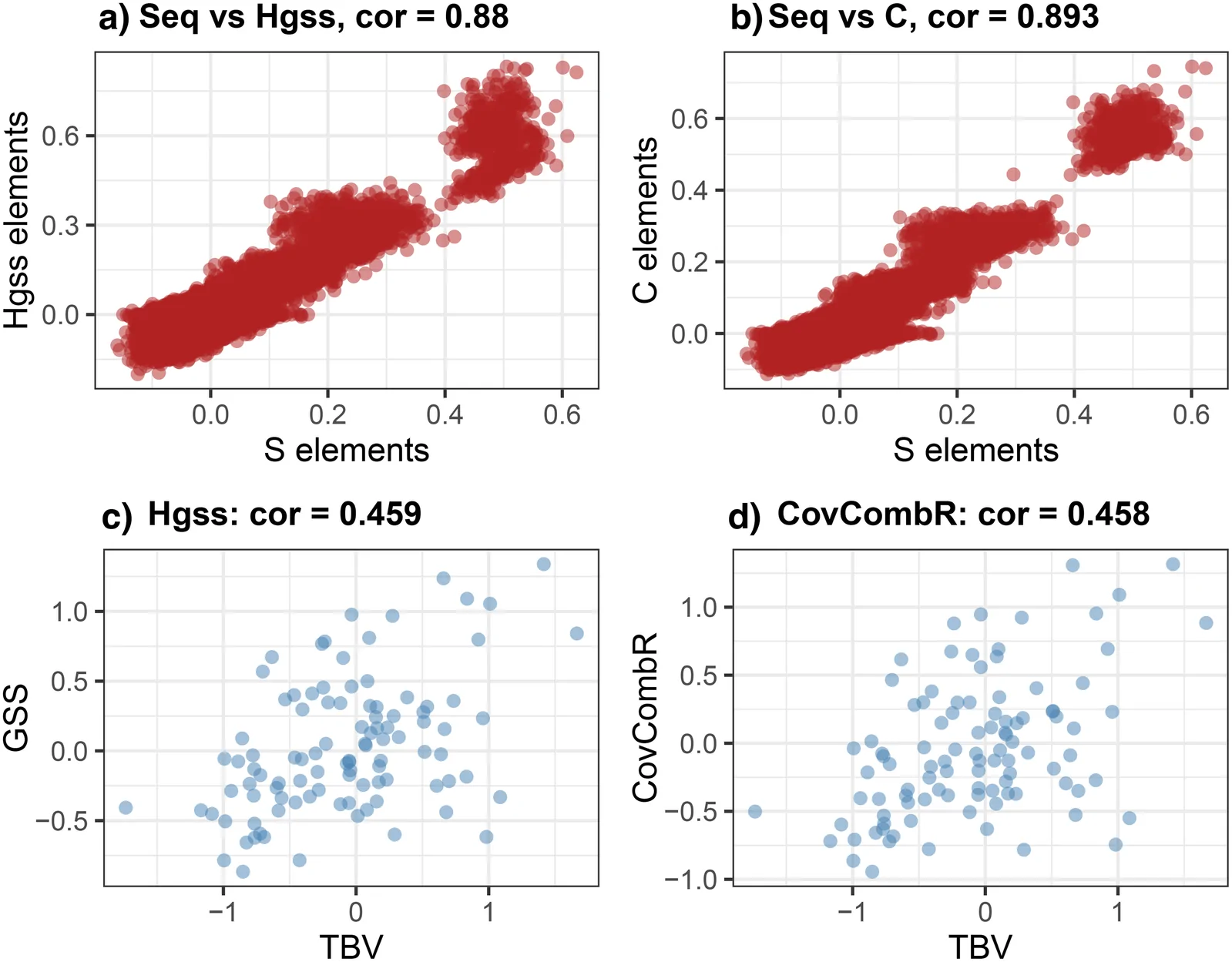
Effect of non-overlapping marker arrays. Legend: (**a**): off-diagonal elements of sequence based GRM (**S**) vs GSS matrix (Hgss); (**b**): off-diagonal elements of sequence based GRM (**S**) vs combined matrix (**C**); (**c**) Correlation between true (TBV) and predicted breeding values when using Hgss in the 100 last generation individuals; (**d**) Correlation between true (TBV) and predicted breeding values when using CovCombR (**C**) matrix. Founder individuals are ungenotyped, generations 1–2 are genotyped with 1k array and generation 3 individuals are genotyped with 15.5k markers

GSS assumes marker arrays are hierarchical, i.e., that all markers in a given array are contained in any array of higher density. In fact, the only assumption required for GSS to hold is *Var*(**u**|**P, M**_**1**_, **… , M**_**i**_) ~ *Var*(**u**|**M**_**i**_), where **M** marker sets are ordered by increasing density. Akdemir et al. [6] proposed an iterative method (CovCombR) to ‘bend’ several relationship matrices towards a ‘consensus’ **C** matrix even if they partially overlap or contain heterogeneous data sources. Due to computing constraints, we compared CovCombR method with GSS in a smaller example where 50% of markers of the low-density 1k SNP array were not included in the highest density array (methods). Figs. 5a, b shows that the correlations between elements of **Hgss** and **C** matrices with the sequence-based matrix were very similar (0.88 vs 0.89, respectively). The predictions with the two methods were comparable in this case (Fig. 5c, d). In a related spirit, Christensen et al. [7] extended SS theory when several independent -omics data are combined with marker information. In the example analyzed, the differences and hierarchy between low- and high-density arrays are obvious (1k vs. 19,5k SNPs). This might not be the always case. Since the requirement of GSS is that *Var*(**u** |**M**_**0**_, **… M**_**i**_) ~ *Var*(**u** |**M**_**i**_), the expected predicted error variances with the different SNP lists could be computed to establish the hierarchy.

In summary, we present a Generalized Single Step (GSS) approach that allows combining an arbitrary number of hierarchical SNP arrays, sequence, known causal loci, and pedigree information in a coherent framework, avoiding successive imputation steps. This note focuses on the theoretical framework and provides the R code that allows in depth analyses of GSS properties in future works, e.g., in comparison to genome wide imputation or other relevant approaches such as that proposed in Christensen et al. [7]. In all, we show that GSS can improve predictive accuracy as new molecular information is added and is much faster than method in [6]. In the case studied, the observed increase with new marker arrays may not be substantial, especially when sequence data is added [15, 16]. In general, we expect a larger influence of sequence data when linkage disequilibrium is small, i.e., effective population size is large, or if a subset of causal mutations can be identified. GSS theory can be used for genetic evaluations in ongoing breeding programs but also to optimize allocation of sequencing and/or genotyping resources, in combination with simulation programs [11, 17]. Finally, although we have developed GSS with animal or plant breeding plans in mind, it is worthwhile noting that GSS may have applications in ecology or human genetics, when pedigree may be not available, but where different sources of molecular data can be combined. Users can test their own choice of parameters with GitHub script (https://github.com/miguelperezenciso/GSS/blob/main/GSS.R).

## Data Availability

Code used to generate content in the mansucript is available at https://github.com/miguelperezenciso/GSS.
